# Tomato Plant Proteins Actively Responding to Fungal Applications and Their Role in Cell Physiology

**DOI:** 10.3389/fphys.2016.00257

**Published:** 2016-06-30

**Authors:** Zoobia Bashir, Sobiya Shafique, Aqeel Ahmad, Shazia Shafique, Nasim A. Yasin, Yaseen Ashraf, Asma Ibrahim, Waheed Akram, Sibgha Noreen

**Affiliations:** ^1^Department of Physics, University of the PunjabLahore, Pakistan; ^2^Institute of Agricultural Sciences, University of the PunjabLahore, Pakistan; ^3^Institute for Medicinal Plants, College of Plant Science and Technology, Huazhong Agricultural UniversityWuhan, China; ^4^Institute of Molecular Biology and Biotechnology, University of LahoreLahore, Pakistan; ^5^Institute of Pure and Applied Biology, Bahauddin Zakariya UniversityMultan, Pakistan

**Keywords:** defense related proteins, matrix plot, proteomic profile, small inhibitory RNA

## Abstract

The pattern of protein induction in tomato plants has been investigated after the applications of pathogenic and non-pathogenic fungal species. Moreover, particular roles of the most active protein against biological applications were also determined using chromatographic techniques. *Alternaria alternata* and *Penicillium oxalicum* were applied as a pathogenic and non-pathogenic fungal species, respectively. Protein profile analysis revealed that a five protein species (i.e., protein 1, 6, 10, 12, and 13) possessed completely coupled interaction with non-pathogenic inducer application (*P. oxalicum*). However, three protein species (i.e., 10, 12, and 14) recorded a strong positive interaction with both fungal species. Protein 14 exhibited the maximum interaction with fungal applications, and its role in plant metabolism was studied after its identification as protein Q9M1W6. It was determined that protein Q1M1W6 was involved in guaiacyl lignin biosynthesis, and its inhibition increased the coumarin contents in tomato plants. Moreover, it was also observed that the protein Q9M1W6 takes significant part in the biosynthesis of jasmonic acid and Indole acetic acid contents, which are defense and growth factors of tomato plants. The study will help investigators to design fundamental rules of plant proteins affecting cell physiology under the influence of external fungal applications.

## Introduction

Plant cell responses under the influence of pathogenic and non-pathogenic fungal species have always been an important aspect in the field of cell biology. These cell responses have also been frequently used in plant protection programs against biotic and abiotic stresses (De Cal et al., 1997; Muniz and Nombela, 2009). It has been studied to manage plant diseases caused by viruses, bacteria and fungal pathogens (Santamarina et al., 2002; Sabuquillo et al., 2005; Islam et al., 2007; Perlin, 2009). All these studies have detected changes in biochemical profiles and protein profiles of plant cells after the application of fungal species. However, only some studies have mentioned that fungal applications change the pathogenesis related (PR) protein profiles of host (Barakat et al., 2012; Khan et al., 2012). It is important to understand alterations in PR protein profiles after application of a pathogenic and a non-pathogenic fungal species to map out the type of changes in plant defense responses under the attack of the fungi (Turner et al., 2002; Chen et al., 2009; Kiba et al., 2012).

Tomato has always been ranked in the list of widely consumed vegetables around the globe. Its dietary uses are very diverse in nature and ranged from domestic to industrial level. Its nutritional value also makes it prominent food item. All of its features have attracted the attention of researchers to improve its yield and quality. Many researchers have investigated the defense weapons of tomato under stress conditions. These studies were conducted to improve tomato yields and to augment tomato cultivation against the activity of a number of its pathogens (Kavroulakis et al., 2006; Girling et al., 2008; Hayat et al., 2010). Among all the pathogens of tomato crop, fungal pathogens are very important due to heavy economic losses caused by them (Shatters et al., 2006). Moreover, *Alternaria alternata* also possesses a distinct position among the list of fungal pathogens of tomato due to its multi types infections at all stages of plant development and crop harvesting. Therefore, experts have focused for the management of *A. alternata* infection on tomato crop using elevated states of plant defenses (induced systemic resistance, ISR) (Shatters et al., 2006; Balestrazzi et al., 2011; Kumar et al., 2012). However, there is a dearth of studies to evaluate the defense related proteins and their roles in cell physiology and controlling fungal pathogens, e.g., *A. alternata* (Hadar and Papadopoulou, 2012). Current investigation has been designed to identify defense related proteins of tomato and their role in cell physiology. Initially, proteins were evaluated for their affinity with non-pathogenic and pathogenic (i.e., *P. oxalicum* and *A. alternata*) fungal species. It will help to understand the PR proteins and their roles in cell physiological processes and ultimately plant defenses. Furthermore, it will also help to understand the nature of plant responses against fungal applications.

## Methodology

### Procurement of tomato cultivars and fungal species

Preliminary studies were performed to screen out tomato cultivars with extremely variable innate resistance against *A. alternata*. Two cultivars Dinaar and Red-Tara were selected with the highest and the least innate resistance, respectively (Ahmad et al., 2013). Afterwards, those cultivars were checked for their innate PR protein profiles in another study, and four protein species were found extra in resistant cultivar, for which results were reported by Ahmad et al. (2014a).

In current investigation both of these cultivars were procured from Fungal Biotechnology Lab, Institute of Agricultural Sciences (IAGS), University of the Punjab (PU), Lahore, Pakistan; and treated with a fungal pathogen (*A. alternata*) and a fungal inducer (*P. oxalicum*). Moreover, two tested fungal species were also procured from the same lab of PU, Lahore, Pakistan. Their cultures were maintained on 2% Malt Extract Agar medium and inocula (3000 spores/mL) were prepared in distilled sterilized water.

### Pot experiment

Seeds of tomato cultivars were grown under greenhouse conditions to minimize adverse environmental fluctuations. Seeds were cultivated in the plastic pots and incubated at 26 ± 2°C. Plants were watered using soil drip irrigation system. At the age of 1 month plants of both cultivars got three treatments, i.e., Pathogen Control “PC” (*A. alternata* only), Inducer Control “IC” (*P. oxalicum* only) and Pathogen+ Inducer “P+I” (*A. alternata*+ *P. oxalicum*). Results of Ahmad et al. (2014a) were taken as Negative Control “NC” (in which the only treatment of distilled sterilized water was applied to tomato cultivars). Inoculum of *A. alternata* was applied in the form of foliar spray on tomato plants by covering soil surface with polythene sheets to avoid unwanted fall of pathogen inoculum on the soil. Inoculum of *P. oxalicum* (100 mL/pot) was applied in the soil. Each treatment of both tomato cultivars was replicated thrice to maintain the reliability of the results; however each replicate consisted upon 10 separate plant pots. After incubation period of 2 weeks, plant samples were analyzed for their protein profiles.

### Protein analysis

#### Extraction

Protein analysis was carried out by adopting the method of Ahmad et al. (2014a). Sodium phosphate buffer saline (140 mM NaCl, 10 mM Na_2_HPO_4_, 1.8 mM NaH_2_PO_4_, and 2.5 KCl) was used to extract total protein contents of plant samples. Protein samples were dissolved in Urea solution (8 M) prior their electrophoration.

#### Separation

Protein samples were elctrophorated on 12% Native Polyacrylamide Gel (Native PAGE). Second dimension of each sample was performed on same gel with the addition of SDS to get fine resolution of protein samples. Then, gel was stained using coomassie blue to record results of visible protein samples. Digital images of protein gels were captured for their detailed analyses.

#### Image analysis

Protein profiles of PC, IC, and P+I of each cultivar were compared with their respective NC protein profiles, and induction index for each protein was calculated with the following formula. A matrix plot showing protein “induction index,” was plotted to evaluate the induction behavior of each protein against the both fungal species.

$$\begin{array}{l} \text{Induction Index}=\sum \text{Ind/Frequency of occurrence}\\ \;\sum \text{Ind}=\text{Number of times a protein was induced by}\\ \text{ fungal inducer }(\text{in comparison to PC}). \end{array}$$

#### Identification

Protein spots were identified and compared using digital software SAMESPOTS (TotalLab Ltd., UK) and TOPSPOT (Kroger and Prehm, Berlin, Germany). Important features of each species were checked from online database UniProt. Solution-state NMR spectroscopy was carried out in order to closely determine physical properties of experimentally important proteins. Data obtained through NMR was statistically analyzed and compared with online protein database using the software PSVS (NorthEast Structural Genomics, NESG).

### Determination of protein roles

The protein showing the highest affinity with tested fungal species was analyzed for its role in cell physiological processes. For this purpose, the synthesis of the protein was blocked using respective small inhibitory RNA. Total metabolites of tomato plants with inhibited synthesis of proteins were extracted and analyzed through gas chromatography mass spectrometery (GCMS) using Clarus SQ8 system of PerkinElmer, USA. Derivatization of samples was carried out by overnight incubating 50 μL plant extract in methoxymine (MOX) reagent (30 μL) at room temperature. Then, 20 μL N-trimethylsilyl N-trifluoroacetamide (MSTFA) was added to each extract sample separately and incubated for 30 min prior to loading of 3 μL sample on GCMS apparatus. Oven temperature was initiated from 70°C and raised up to 200°C with a continuous increment of 5°C/min. Then temperature was raised up to 240°C with continuous increment of 10°C/min. Mass spectrum of each eluted biochemical was recorded during the operation and analyzed by MZmine (Pluskal, Okinawa, Japan).

## Results

### Protein profile analysis

**Figure 1:**
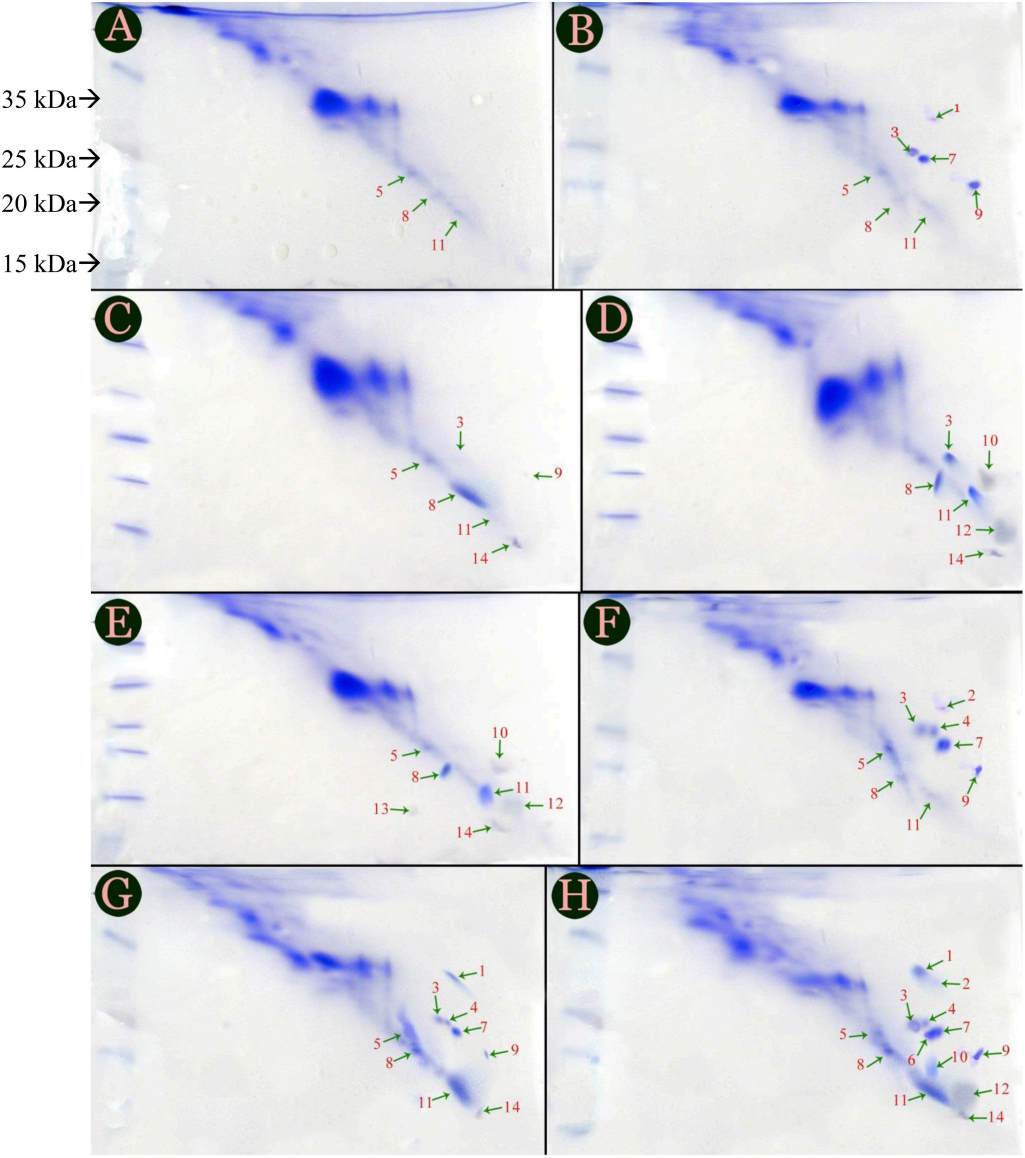
**Protein profile of six treatments given to two cultivars of tomato, i.e., Dinaar (resistant cultivar) and Red Tara (susceptible cultivar)**. Different portions of this figure are **(A)** Negative control of Red Tara; **(B)** Negative control of Dinaar **(C)** Pathogen Control of Red Tara; **(D)** Inducer Control of Red Tara; **(E)** Joint application of Pathogen + Inducer at Red Tara; **(F)** Dinaar treated with pathogen alone; **(G)** Dinaar treated with inducer alone; **(H)** Dinaar subjected to combine application of pathogen + inducer. Negative controls of both cultivars were extracted from Ahmad et al. (2014a).

Protein profiles of tomato cultivars were labeled by streaking images horizontally and confirmed that there were only 6 novel, defense related proteins; but 3 and 7 are presented in Figures 1A,B showing protein profiles of PC and IC treatments of Red Tara cultivar. However, types of proteins synthesized in both the treatments were altered. Protein 9 was present in PC of Red Tara but it was replaced with protein 10, when same plant was inoculated with inducer species. Similarly, protein 5 which had been synthesized in PC of susceptible cultivar was eliminated in IC of same cultivar. However, fungal inducer initiated the biosynthesis of the protein 12 in IC and P+I of Red Tara, which was not previously synthesized in PC. Therefore, production of protein 12 seems to be entirely dependent upon inoculation of *P. oxalicum* (Figures 1B,C). Protein 13 was also found coupled with P+I, as it was synthesized only when Red Tara was treated with P+I. No more combination of treatment and cultivar could produce protein 13 in the study (Figure 1).

PC Dinaar cultivar exhibited eight protein bands which varied both in quantity and quality throughout the treatments provided to the cultivar. However, it was deficient in protein 14 as compared to PC of Red Tara (Figures 1A,D). Three additional proteins in PC Dinaar were protein 2, 4, 7; which were absent in same treatment of Red Tara. Hence, they can be the reason for stronger innate antifungal resistance of Dinaar than Red-Tara. It was also noticed that protein 1 and 14 were triggered by inducer in IC and P+I of resistant cultivar (Figures 1E,F). Meanwhile, P+I inoculation on the resistant cultivar resulted into induction of three proteins, i.e., protein 6, 10, and 12 (Figure 1F).

### Protein identification

A total of 14 protein species were identified from tomato plants. Analysis of these proteins on database declared an association of nine protein species (among the total 14 proteins) had already been proved with *Arabidopsis thaliana*. Rest of the proteins had also plant cell origin. Protein species 4 (Q9LJB7) had been reported from plant cell nucleus and lays its role in blue light pathway. Moreover, it also responds toward detection of chitin. A plant cell mitochondrial protein (Q9M0V0), which plays its role in electron transport chain and biotin synthesis was inversely associated with inducer application. Similarly, a DNA binding protein species 1 (Q9SN83) was also inversely associated with application of the pathogenic fungal species.

Protein species 10 (Q9FJY1) and 12 (Q9LF87) recorded their higher affinities with application of both fungal species (pathogenic and non-pathogenic). Protein 14 (Q9M1W6) exhibited the highest association with all types of fungal applications. It was always 100 percent associated with inducer application, while 50% with *A. alternata* in case of resistant cultivar (Table 1).

**Table 1:** **Protein species showing translational association with microbial applications**.

**Species number**	**Species identifier**	**Group**	**Size (kDa)**	**Function**	**Previously reported in**
1	Q9SN83	Zinc finger superfamily	31.8	Binding of DNA folds	*Arabidopsis thaliana*
2	Q9SMP4	Uncharacterized	30.6	Zinc ion binding	*A. thaliana*
3	Q9LJB7	Transcription regulators	25.1	Blue light signaling pathway Response to chitin	Plant cell nucleus
4	Q9M138	CHP-rich zinc finger protein-like	25.6	Unknown	*A. thaliana*
5	Q9M0V0	Adrenodoxin family	21.8	Biotin biosynthesis Electron carrier in electron transport chain	Plant cell mitochondria
6	Q9ZU56	Uncharacterized	21.6	Unknown	*A. thaliana*
7	O04646	Zim17-type zinc finger protein	23.3	Unknown	*A. thaliana*
8	O81296	Uncharacterized	18.4	Unknown	Vacuolar membrane
9	Q9C851	Zinc ion binding	18.1	Binding of nucleic acid	*A. thaliana*
10	Q9FJY1	Antigen 5	20.1	Cysteine-rich secretory proteins	*A. thaliana*
11	Q9LZV8	Zinc ion binding	16.8	Protein modification and ubiquitination	Single-pass membrane protein
12	Q9LF87	Cabohydrate binding	15.5	D-galactoside/L-rhamnose binding SUEL lectin protein	*A. thaliana*
13	Q9LX43	Uncharacterized	13.3	Protein import into mitochondrial matrix	Mitochondrial inner membrane presequence translocase complex
14	Q9M1W6	Uncharacterized	12.9	Unknown	*A. thaliana*

Fourteen protein species have been enlisted with their respective identifiers, family functions and sizes.

Protein 14 (Q9M1W6) was strictly associated with inducer application in both of the tested cultivars. However, protein species 10 and 12 were completely inducer associated in case of susceptible cultivar, but their association decreased to 50% in case of resistant cultivar. Furthermore, Q9M138 (protein 4) showed completely positive relation with all fungal applications in case of resistant cultivar Dinaar; however, it did not show any response in case of susceptible cultivar. Interestingly, there were only two proteins (2 and 4), which were associated significantly with the pathogen in resistant cultivar, and the same proteins did not respond to pathogenic application in susceptible cultivar.

**Figure 2:**
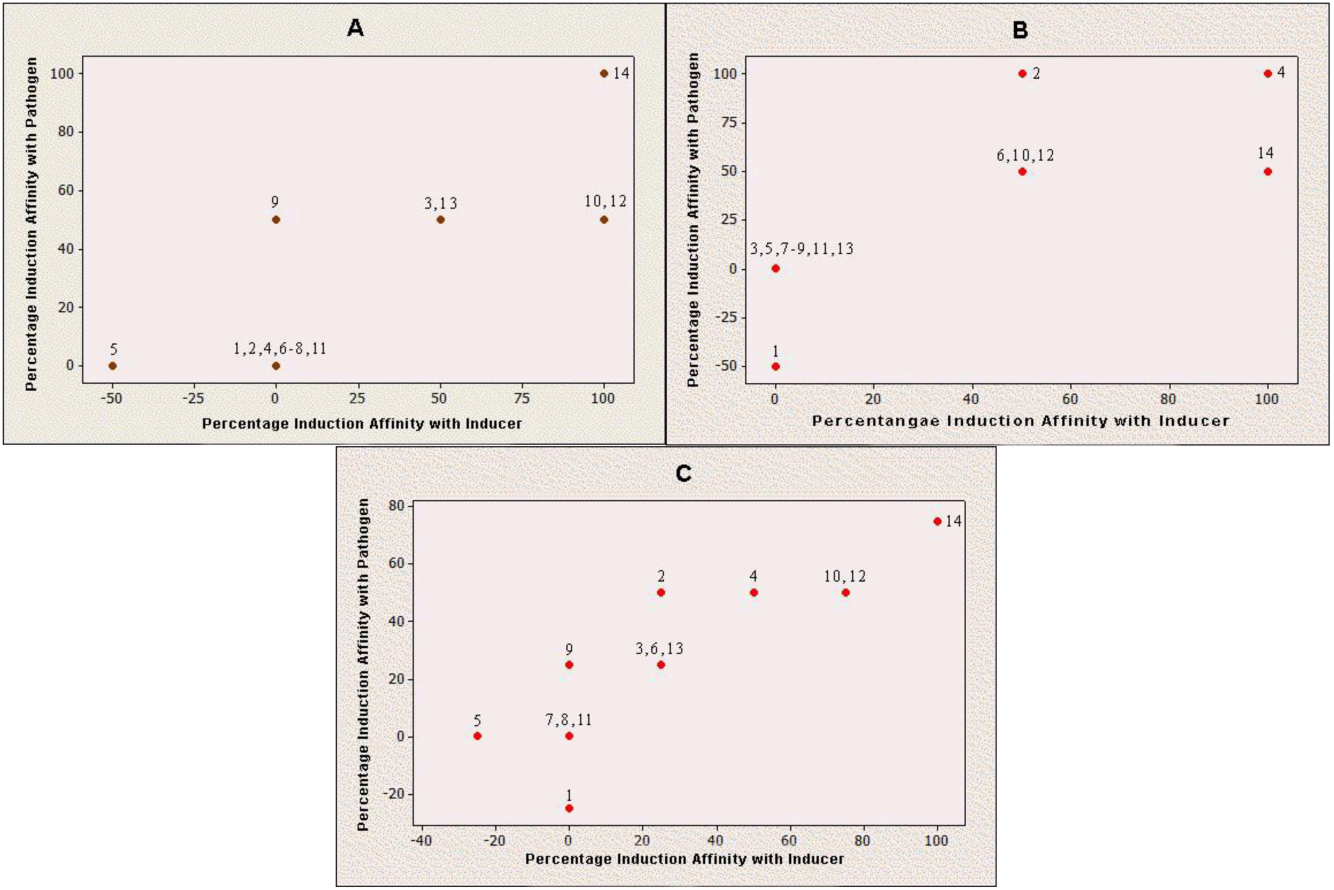
**Induction behavior of individual proteins against *Penicillium oxalicum***. Positive value indicates that production of a particular protein is triggered by fungal inducer and negative value means that inducer has counter acted with respective protein production; while, zero value reveals inert behavior of protein for *P. oxalicum*. **(A)** Induced protein profile of Red Tara (susceptible cultivar); **(B)** Induced protein profile of Dinaar (resistant cultivar); and **(C)** Average protein induction profile of tomato cultivars.

Production of Q9M0V0 (protein 5) was inhibited after inducer applications in susceptible cultivar. However, the same protein did not respond toward any fungal application in resistant cultivar. On the other side, protein 1 interacted negatively with pathogen application in case of resistant cultivar, while the same protein species did not respond to any fungal species in case of susceptible cultivar. It was also noticeable that the interactions of all negatively behaving proteins were partial and no protein showed 100% negative behavior with any fungal application. Furthermore, Q9M1W6 was the only protein which exhibited 100% positive interaction with pathogenic applications on susceptible cultivar (Figure 2).

**Figure 3:**
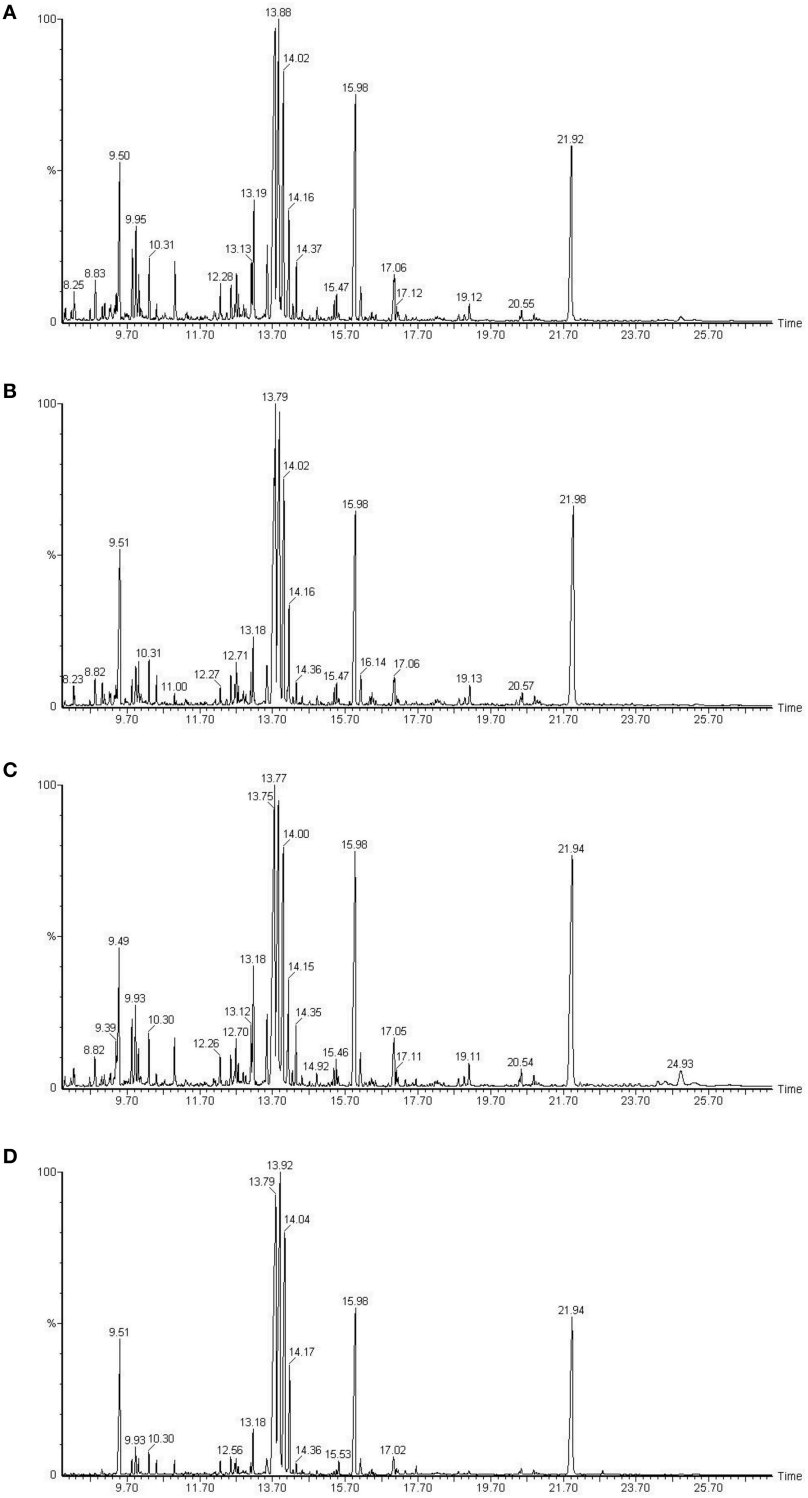
**Gas chromatography mass spectrometric analysis of two different tomato cultivars, Dinaar (resistant cultivar) and Red Tara (susceptible cultivar)**. The sections **(A,C)** compare total metabolites of Dinaar and Red Tara with normal protein profile, respectively. However, the sections **(B,D)** represent the total metabolite analysis of Dinaar and Red Tara, respectively, after the inhibition of protein 14 (Q9M1W6). Elution time of biochemicals has been mentioned along X-axis, however peak height along Y-axis represents the quantity of eluting biochemical. Significant peaks have also been separately labeled with their elution time in plot area.

Chromatogram of control plants of cultivar Dinaar exhibited more peak heights elevated than the plants with inhibited protein species. Biochemical eluting at 10.31 min of chromatographic run had increased peak height in control treatment of Dinaar cultivar. The similar trend was also observed in case of Red Tara cultivar, in which control treatment plants showed more peak heights as well as more number of peaks than the respective inhibited protein plants (Figure 3).

Deoxygluconate recorded the highest fold change in its quantity due to the inhibition of protein 14 (Q9M1W6). Retionic acid was increased up to 4.16 and Pentadecanoic acid also showed an increase of 3.76-folds after the inhibition of the protein. Sugar contents, e.g., Fructose, Galactopyranose, Galactose, and L-altropyranose recorded significant increase in their contents (1.66, 1.2, 2.87, and 3.56-folds, respectively). The sugar with minimum increase in its contents (0.31) after the protein inhibition was Rhamnose. Some acid contents of tomato metabolites (Aminobutyric acid, Capric acid, Carboxylic acid, Acetic acid, and Glutaminic acid) also showed an increase after inhibition of the protein. However, contents of Hepatonic acid, Sinapic acid, Jasmonic acid, Ferulic acid, Quinic acid, and Xylonic acid were decreased up to −3.54, −2.05, −1.76, −2.88, −2.64, and −1.58-folds, respectively. Moreover, Indole acetic acid recorded a decrease of −0.47-folds and p-Coumaric acid reduced up to −0.82-folds in the plants with inhibited protein Q9M1W6 (Table 2).

**Table 2:** **Heat map of biochemicals showing significant fold changes after inhibition of protein 14 (Q9M1W6)**.

**Chemical name**	**Fold change**	**Chemical name**	**Fold change**
Acetic acid	1.42	Aminobutyric acid	1.75
Alpha-Phocaecholic acid	0.68	Capric acid	0.82
Fructose	1.66	Carboxylic acid	2.21
Galactopyranose	1.2	Glucose	1.23
Galactose	2.87	L-altropyranose	3.56
Deoxygluconate	4.51	Glutaric acid	2.51
Dichloro acetate	2.19	Heptonic acid	–3.54
Glutaminic acid	2.34	Manopyranose	2.98
Cinnamate	–1.35	Coumarinate	–0.38
L-Arabinopyranose	–0.49	Rhamnose	0.31
Lyxose	0.37	Hexadecanoic Acid	–2.96
Mannose	2.61	Hexanoic acid	–2.62
Hydrocortisone succinate	–4.34	Hexonic acid	–3.88
Indole acetic acid	–0.47	L-Threonate	1.23
N-Acetylneuraminic acid	1.64	Malic acid	1.82
Nonanoic acid	2.14	Oxobutyric acid	2.54
Pentadecanoic acid	3.76	Oxodecanoate	2.79
Pentonic acid	3.68	Oxohexanoic acid	–0.98
p-Coumaric acid	–0.82	Sinapyl alcohol	1.43
Caffeic acid	3.41	Sinapic acid	–2.05
Ferulic acid	–2.88	Coniferyl alcohol	0.67
Quinic acid	–0.64	Retionic acid	4.16
Jasmonic acid	–1.76	Ribonic acid	1.41
Shikimic acid	1.74	Sulfoacetate	–0.43
Xylonic acid	–1.58		

**Figure 4:**
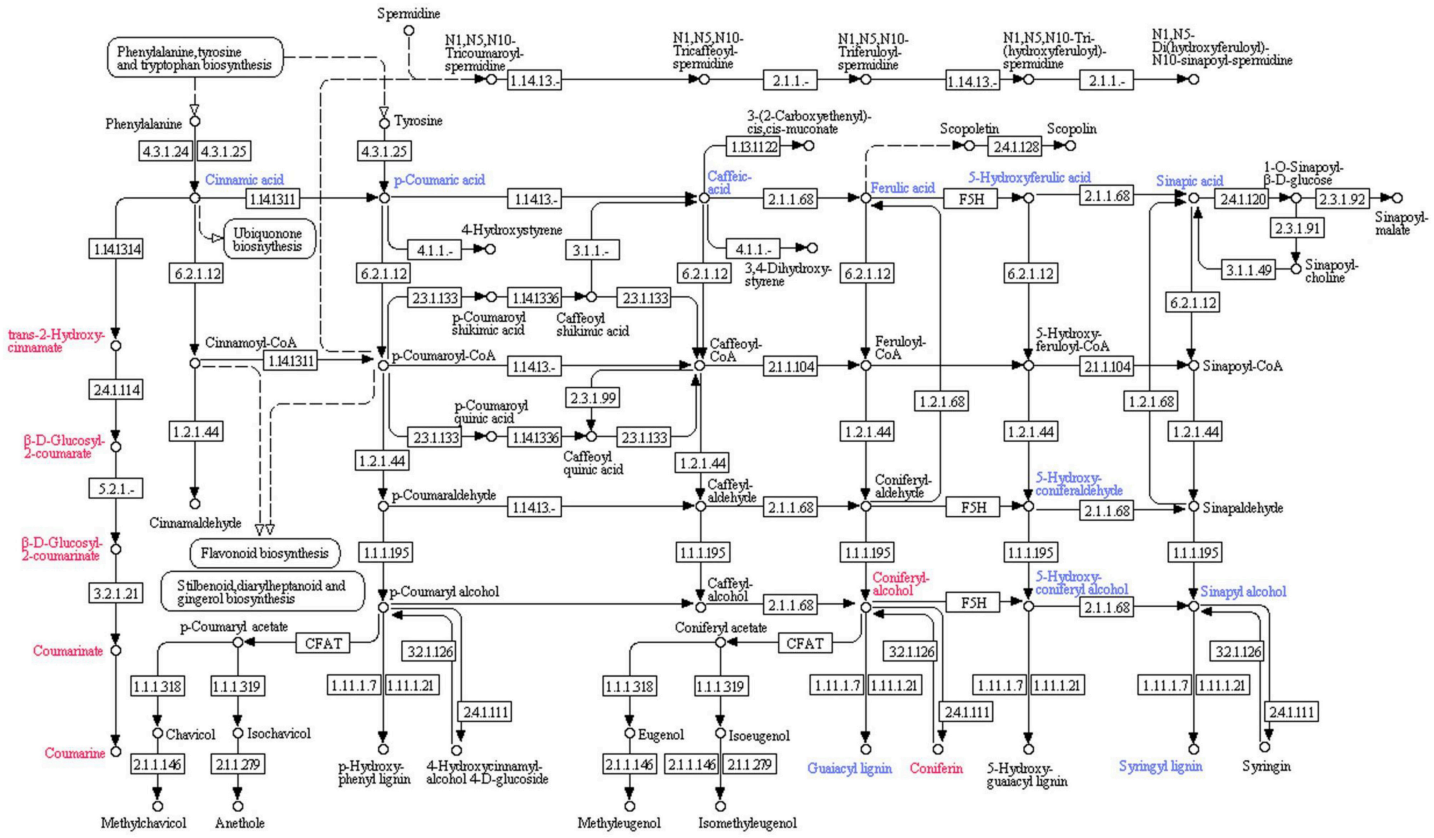
**Effect of inhibition of protein 14 (Q9M1W6) on metabolism of tomato plant**. Hierarchy of biochemical involved in plant metabolism was plotted with the help of KEGG pathway database. Compounds shown in red color were found to be enhanced after inhibition of the protein species. However, blue color represents compounds with reduced quantities after inhibition of the protein. Numerics have been provided to show related enzyme catalyzing specific reactions according to the Kyoto Encyclopedia of Genes and Genomes (KEGG) enzyme library.

Inhibition of protein 14 (Q9M1W6) resulted in the retardation of cinnamic acid contents. The decreased cinnamic acid contents triggered a chain of organic acids with reduced quantities (i.e., p-Coumaric acid, caffeic acid, ferulic acid, 5-Hydroxyferulic acid and sinapic acid). Those induced organic acids boosted the quantities of downstream biochemicals in their pathways. Coniferyl alcohol was recorded to be enhanced by ferulic acid in the downstream process of coniferin and guaiacyl lignin biosynthesis. Coniferin contents were found to be increased due to enhanced coniferyl alcohol contents. However, a reduction in the quantity of guaiacyl lignin was recorded. Decreased contents of 5-Hydroxyferulic acid resulted into reduced quantities of 5-Hydroxyconiferaldehyde, which also retarded 5-Hydroxy-coniferyl alcohol in tomato plant tissues. However, it didn't induce 5-Hydroxyguiacyl alcohol (a descending biochemical in its pathway) and its quantity didn't show any significant change. Decrease in sinapic acid quantities also reduced sinapyl alcohol contents in plant tissues. It was also notable that 5-Hydroxy-coniferyl alcohol was also involved in reduction of sinapyl alcohol contents. Therefore, sinapyl alcohol and its downstream biochemical syringyl lignin showed reduced quantities (Figure 4).

Inhibited protein 14 resulted into the enhanced quantities of trans-2-Hydroxy cinnamate, which is an intermediary biochemical in the biosynthesis of coumarins. Moreover, it started the chain of biochemicals with increased quantities, i.e., β-D-Glucosyl-2-Coumarate and β-D-Glucosyl-2-Coumarinate, which are the intermediates in coumarin biosynthesis. The chain proceeded toward coumarinate, a precursor of coumarin; and ended at the elevated coumarin contents (Figure 4).

## Discussion

Inoculation of plant roots with a biotic inducer may modulate plant resistance in the above ground tissues against a number of pathogens (Pieterse et al., 2002). That boosted resistance has a true involvement of alterations in protein profile of plant (Maleck et al., 2000). Therefore, whenever a plant faces enhanced state of resistance, it is actually producing an altered protein profile actually. Present study also affirms this phenomenon because protein profiles had been entirely deviated from the normal. Plants who were successful in avoiding pathogen infection must have synthesized defense related proteins in their cells. Therefore, protein for which high biosynthesis was recorded in induced tissues were definitely resistance conferring proteins. Those proteins can also be recorded in a database, and can also be used for making a plant resistant against fungal pathogens, when it is needed.

A function toward chitin synthesis had already been reported for protein 4 (Q9LJB7), which clearly indicates its role against invading fungal pathogens (Kaneko et al., 2000; Libault et al., 2007). Interestingly, Q9LJB7 was recorded highly associated with fungal applications on resistant tomato cultivar Dinaar. It can be assumed that the strong antifungal resistance of Dinaar was due to the higher biosynthesis of Q9LJB7. However, the biosynthesis behavior of a DNA binding protein species 1 (Q9SN83) was recorded totally opposite to the Q9LJB7, because production of Q9SN83 was hindered upon application of pathogenic fungal species. It can be supported with the higher transcriptional rates of plant cells during the pathogenic attacks, which would definitely need uncoiling of DNA strands (Ahmad et al., 2014a; Akram et al., 2014). Therefore, the requirement of DNA binding proteins was automatically retarded during the phase of higher transcriptions in the cells. Due to which, protein Q9SN83 showed inverse relations with the application of the fungal pathogen (*A. alternata*).

Q9FJY1 and Q9LF87 were found to be highly associated with fungal applications on tomato plants. The reported function of Q9FJY1 is to facilitate production of cysteine rich secretory proteins; while, Q9LF87 had been involved in the binding of carbohydrates especially D-galactoside/L-rhamnose (Tabata et al., 2000a,b). This indicates that tomato plants give more stress upon efficient secretory system of their cells during fungal attack.

Protein species exhibited maximum association with fungal applications (Protein 14) had not been previously characterized for its functions. However, this study concludes it as a very important protein with respect to fungal pathogens and plant protection program. However, characterization of this protein, determination of its functions and its categorization in the most suitable group need more scientific investigations to be done.

Moreover, results indicate that Penicillium induced the production of protein 2 and protein 4 in resistant cultivar, which can be linked with induced systemic resistance (ISR). However, Penicillium did not elevate the production of these proteins in susceptible cultivar; rather than it inhibited the production of protein 5. It clearly indicates that mechanism of ISR differs with respect to the level of innate resistance of the cultivar. Ahmad et al. (2014b) also pointed out that nature of ISR was different with respect to the cultivar of tomato. They concluded that ISR elevated the rate of salicylic acid pathway in the cultivars with higher levels of innate resistance; however, it decreased the rate of jasmonic acid pathway in case of susceptible cultivars. The current investigation also concludes more or less similar results on the basis of protein profiles. It can be concluded here that ISR is due to elevation of protein 2 and 4 in resistant cultivar; and due to inhibition of protein 5 in case of susceptible cultivar.

Extensive work by Mendoza-Mendoza et al. (2003) and Pozo et al. (2004) explored that some types of lytic enzymes are released by fungal species. These enzymes are detected by plants and concomitantly plants modify their metabolism to cope with expected fungal infection. This also results into suppression or over-production of plant defensive proteins. Therefore, present study falls in agreement with previously described investigation as fungal inoculum modulated plant protein profile remarkably. Some studies also conclude that induction of proteins make a plant highly resistant against fungal pathogens. Sometimes, these proteins are lytic enzymes in nature which degenerate pathogen growth and disturb its metabolism as well (Emani et al., 2003). Therefore, it is obvious that if plant gains antifungal resistance then it not only produce increased amounts of existing proteins, but it may also synthesize new types of proteins. Similarly, present study discovered five protein species which were entirely in direct relation with the inducer species. Resistance induction does not means that plant starts producing heavy amounts of defense proteins and directs its energy budget toward anti-pathogenic activities. However, it means that a plant with induced resistance may quickly and efficiently produce defensive elements on being infected with a challenging pathogen (Conrath et al., 2002). Present investigation falls in agreement with this phenomenon because induced plants have higher amounts of proteins only in the presence of pathogen.

Plant resistance induced by a non-pathogenic biotic inducer and by a pathogenic entity are mediated by different signaling pathways (Pieterse et al., 1998; Whipps, 2001). Hence, there may some relevance among the end biochemicals induced in two cases; but, it is not mandatory. It's possible for an inducer and pathogen that they trigger different types of resistance factors in a plant, or they may stimulate biosynthesis of similar defenses also. All of these metabolic interactions always result into complex outputs.

The study explored the response of a protein (Q9M1W6) after the application of fungal species, and its role in modulation of plant metabolism. It has been clarified from the results that the protein was involved in the regulation of lignin biosynthesis. Its inhibition significantly reduced the quantities of lignin and most of the intermediates of lignin biosynthesis pathway. Moreover, some of the intermediates, e.g., coniferyl alcohol was found to be enhanced; however, it produced the elevated quantities of coniferin and couldn't yield guaiacyl lignin. It is notable that coniferyl alcohol is the intermediate for the biosynthesis of guaiacyl lignin and coniferin. There is a need of more investigations to explore the factors which modulated higher coniferyl alcohol contents to produce increased coniferin only. It is clear from the recorded data that after induction from fungal species, the protein enhances lignin accumulation in plant cell walls, which may enhance plant defenses against the fungal pathogens (Ahmad et al., 2014c).

A decrease in jasmonic acid contents was recorded in current investigation, which is an important defense element in tomato plants (Pieterse et al., 1998; Whipps, 2001; Conrath et al., 2002; Emani et al., 2003; Mendoza-Mendoza et al., 2003; Pozo et al., 2004; Ahmad et al., 2014b,c). Its decreased contents indicate the reduced defense responses of plants against pathogen attacks. Therefore, tomato pants plants inhibited or deficient in protein Q9M1W6 are not suitable for cultivation in agricultural farms due to high risk of disease attacks. Moreover, an inhibition of protein Q9M1W6 also resulted into reduced Indole acetic acid contents, which is a clear indication of the dwarf growth of tomato plants. However, this effect of the inhibited protein Q9M1W6 can be utilized to achieve special goals related to plant height.

The inhibition of the protein (Q9M1W6) also resulted into elevated contents of coumarins and its intermediates. Coumarin contents play an important role in defining palatability of food. Enhanced coumarin contents represent the increased palatability of the food items (Blahová and Svobodová, 2012). Keeping in mind coumarin contents and palatability, current investigation can be concluded as the inhibition of protein may increase the palatability of tomatoes. However, it enhances plant defense against pathogens by strengthening plant cell wall due to accumulation of lignin.

Altogether, proteins are themselves are the most active defense weapons of the plant cells, and they may also drive the other defense responses of the plants (Zhu et al., 2010; Cândido Ede et al., 2011). The actual function of a cell or tissue is basically dependent upon its protein profile (Pontén et al., 2009). Moreover, the level of antifungal resistance in plants actually depends upon modifications in protein profile of plant cells.

## Author contributions

NY, SoS, and ShS conceived the idea of this manuscript. AA, YA, SN, and WA designed this study. ZB, AA, and AI performed the experiment. NY, YA, ShS, SN, and SoS record and analyzed the data. ZB, AI, and WA wrote this manuscript. All the authors reviewed the manuscript and recommended its submission.

### Conflict of interest statement

The authors declare that the research was conducted in the absence of any commercial or financial relationships that could be construed as a potential conflict of interest.
